# *Ehrlichia* Species in Dromedary Camels (*Camelus dromedarius*) and Ruminants from Somalia

**DOI:** 10.3390/pathogens14010065

**Published:** 2025-01-13

**Authors:** Aamir M. Osman, Ahmed A. Hassan-Kadle, Marcos R. André, Flávia C. M. Collere, Amir Salvador Alabí Córdova, Fabiano Montiani-Ferreira, Thállitha S. W. J. Vieira, Abdalla M. Ibrahim, Abdulkarim A. Yusuf, Rosangela Z. Machado, Rafael F. C. Vieira

**Affiliations:** 1 Vector-Borne Diseases Laboratory, Department of Veterinary Medicine, Universidade Federal do Paraná, Curitiba 80035-050, Brazil; flaviacollere@gmail.com (F.C.M.C.); montiani@ufpr.br (F.M.-F.); 2 Somali One Health Centre, Abrar University, Mogadishu P.O. Box 25, Somalia; akadle@abrar.edu.so (A.A.H.-K.); abdallami@abrar.edu.so (A.M.I.); 3 Department of Animal Health and Veterinary Services, Ministry of Livestock, Forestry, and Range, Mogadishu, Somalia; 4 Abrar Research and Training Centre, Abrar University, Mogadishu P.O. Box 25, Somalia; karimvet@abrar.edu.so; 5 Vector-Borne Bioagents Laboratory (VBBL), Department of Pathology, Reproduction and One Health, Faculty of Agrarian and Veterinary Sciences, São Paulo State University (FCAV/UNESP), Jaboticabal CEP 14884-900, Brazil; mr.andre@unesp.br (M.R.A.); amir_cordova@hotmail.com (A.S.A.C.); 6 Department of Veterinary Medicine, Universidade Federal do Paraná, Curitiba 80035-050, Brazil; 7 Department of Chemistry, The University of North Carolina at Charlotte, Charlotte, NC 28223, USA; t.vieira@charlotte.edu; 8 Department of Slaughterhouses, Somali Meat Company, Mogadishu, Somalia; 9 Center for Computational Intelligence to Predict Health and Environmental Risks (CIPHER), The University of North Carolina at Charlotte, Charlotte, NC 28223, USA; 10 Department of Epidemiology and Community Health, The University of North Carolina at Charlotte, Charlotte, NC 28223, USA

**Keywords:** tick-borne diseases, ehrlichiosis, *Ehrlichia minasensis*, *Ehrlichia ruminantium*, heartwater, Somalia, sub-Saharan Africa

## Abstract

Ehrlichioses, caused by *Ehrlichia* species, are tick-borne diseases (TBDs) that affect animals and humans worldwide. This study aimed to investigate the molecular occurrence of *Ehrlichia* spp. in 530 animals (155 Dromedary camels, 199 goats, 131 cattle, and 45 sheep) in the Benadir and Lower Shabelle regions of Somalia. Blood DNA samples were tested for PCR targeting *dsb* and *sodB* genes of *Ehrlichia* spp. and *PCS20* and *map1* genes of *E. ruminantium*. The obtained sequences were submitted for phylogenetic analyses. *Ehrlichia* spp. were detected in 26.4% (140/530) of animals by *dsb*-PCR, with the highest prevalence in dromedary camels (54.8%), followed by cattle (29.8%), goats (7.0%), and sheep (4.4%). Dromedary camels, cattle, and goats had significantly higher infection odds compared to sheep (*p* < 0.05). Among *dsb*-PCR-positive samples, 76.9% (30/39) of cattle tested *sodB*-positive, while other species were negative. *E. ruminantium* was detected in 13.7% (18/131) of cattle by *pCS20*-PCR, but none were positive for the *map1* gene. Phylogenetic analysis confirmed *E. minasensis* in camels, sheep, and goats and *E. ruminantium* in cattle, marking the first molecular evidence of *E. minasensis* in dromedary camels, sheep, and goats globally, and *E. ruminantium* in cattle from Somalia. These findings emphasize the need for further research on its economic and public health impact.

## 1. Introduction

Ehrlichioses are tick-borne diseases (TBDs) caused by at least six bacterial species of the genus *Ehrlichia*, namely *E. canis*, *E. chaffeensis*, *E. ewingii*, *E. muris*, *E. ruminantium*, and *E. minasensis* [1,2]. These diseases affect animals and humans worldwide, representing a growing public health concern [3].

Heartwater disease, caused by *E. ruminantium*, is a notifiable disease that is listed by the World Organization for Animal Health (WOAH) [4]. It is primarily transmitted by *Amblyomma* ticks and affects domestic and wild ruminants across sub-Saharan Africa and Caribbean islands. Goats and sheep are more susceptible than cattle, posing significant economic concerns [2,4,5]. Therefore, comprehensive attention should be given to its impact on both wildlife and domestic livestock.

Livestock play a significant economic role in Somalia as they serve as a vital source of food, a form of currency, and a means of generating export earnings. In the Lower Shabelle region, the predominant farming system is the traditional mixed-farming system. However, the development of livestock in these systems has been hindered by various factors, including TBDs [6]. Additionally, challenges such as poor livestock nutrition and management practices, as well as the lack of tailored disease control strategies that are adapted to the unique characteristics of these systems, further contribute to the limitations faced in livestock development. Addressing these challenges and implementing effective disease control measures are crucial for improving the overall health and productivity of livestock in Somalia’s traditional mixed farming systems.

Globally, ehrlichioses have been extensively studied in dogs, but they have been mostly neglected in other animal species. In ruminants and dromedary camels, few studies have described the presence of *Ehrlichia* spp. Notable instances include cattle from Canada (*Ehrlichia* spp.) [7], Brazil (*E. minasensis*) [8,9,10], France (*E. minasensis*) [11], Pakistan (*Ehrlichia* sp. (Multan) and *E. minasensis*) [12], Ethiopia (*E. ruminantium* and *E. minasensis*) [13], South Africa (*E. canis* and *Ehrlichia* spp.) [14], and Kenya (*E. minasensis)* [15]. Additionally, small ruminants from the Caribbean islands (*E. canis)* [16], China (*E. canis* and *Ehrlichia* spp.) [17], Kenya, Gambia, Senegal, South Africa, Sudan, Uganda, and Malawi (*E. ruminantium*) [18], Italy (*E. canis*) [19], and Malawi (*E. canis*) [20], as well as dromedary camels from Kenya (‘*Candidatus* Ehrlichia regneryi’; *E. ruminantium*) [21,22] and Saudi Arabia (*E. canis* related strains) [23], have also been identified as hosts for *Ehrlichia* spp.

The distribution of ehrlichiosis is related to the environmental requirements of the tick vectors [24]. Molecular evolution analysis suggests that *E. minasensis* likely originated from highly variable strains of *E. canis* [1], and it has been reported in various locations, including Canada [7], Brazil [8], France [11], Pakistan [12], Ethiopia [13], South Africa [14], and Kenya [15]. It has also been detected in several tick species, including *Rhipicephalus* sp., *Hyalomma* sp., and *Amblyomma* sp., suggesting that several tick species may vector this agent [11,12,25,26]. In Somalia, six tick species, namely *Rhipicephalus pulchellus*, *Hyaloma truncatum*, *Hyaloma dromedarii*, *Ambyomma gemma*, *Ambyomma lepidum*, and *Ambyomma variegatum*, have been commonly identified and widespread [27,28]. However, no survey on *Ehrlichia* infection has been reported to date. Accordingly, this study aimed to perform a comprehensive molecular survey for *Ehrlichia* sp. in ruminants and dromedary camels from Somalia.

## 2. Material and Methods

### 2.1. Study Area and Design

A cross-sectional study was carried out between December 2018 and March 2022 in the Benadir (2.1065° N, 45.3933° E) and Lower Shabelle Regions (1.8670° N, 44.5502° E), Somalia. For this purpose, a non-probabilistic convenience sampling was performed. A total of 131 cattle (*Bos taurus indicus*), 199 goats *(Capra aegagrus hircus*), 45 sheep (*Ovis aries*), and 155 dromedary (*Camelus dromedarius*) blood samples were collected by jugular venipuncture. Animals were physically restrained, and blood samples (5 mL) were collected by venipuncture of the jugular vein using vacuum tubes containing EDTA (BD Vacutainer^®^, Franklin Lakes, NJ, USA). A total of 125 µL of blood samples from 104 dromedaries, 199 goats, and 45 sheep were applied on filter paper (FTA^®^ card, Whatman^®^, GE Healthcare, Buckinghamshire, United Kingdom), while EDTA blood samples from 131 cattle and 51 dromedaries were collected and stored at −20 °C until molecular analysis. Samples were retrieved from previous studies [28,29].

### 2.2. DNA Extraction

DNA was extracted from dried blood spots (DBS) on filter paper (FTA^®^ card, Whatman^®^, GE Healthcare, Buckinghamshire, United Kingdom) from 104 dromedary camels, 199 goats, and 45 sheep, and from 200 μL of whole blood from 131 cattle and 51 dromedary camels using a commercial kit (IndiMag^®^ Pathogen Kit, Qiagen for Indical Bioscience, Leipzig, Germany), according to the manufacturer’s instructions.

For DBS, three discs were removed from each sample using a three mm Punch (Harris Uni-Core™, Qiagen, Hilden, Germany) and placed into clean RNase/DNase-free 1.5 mL microtubes. Pre-treatment was performed by adding 200 µL of Phosphate-buffered saline (PBS) to the macerated FTA discs using an 18-gauge needle and heating for 10 min at 96 °C. Filter paper discs without blood and RNase/DNase-free water were used in parallel as a negative control to monitor cross-contamination. DNA samples were stored at −20 °C before PCR assays. 

EDTA blood samples from 131 cattle and 51 dromedaries stored in DNase/RNase-free microtubes were subjected to DNA extraction using the aforementioned commercial kit, according to the manufacturer’s recommendations. Ultra-pure water was used as negative controls to monitor cross-contamination in each batch of extraction.

### 2.3. Polymerase Chain Reactions (PCR) Assays

Conventional PCR for the mammal endogenous gene glyceraldehyde-3-phosphate dehydrogenase (*gapdh*) was performed in all samples to monitor DNA extraction [30]. DNA samples were initially screened by a nested-PCR assay based on the 349 bp of the disulfide bond formation protein gene (*dsb*) gene from *Ehrlichia* spp., as previously described [31]. Additionally, to further molecularly characterize *Ehrlichia* spp., PCR-positive samples were subjected to a PCR assay targeting 300 bp of the *sodB* gene [32]. DNA of *E. canis* (Jaboticabal strain) was used as a positive control, and ultrapure nuclease-free water was used as a negative control in all analyses.

The screening for *E. ruminantium* for cattle DNA was performed using a semi-nested PCR targeting a fragment of 280 bp of ribonuclease III (*pCS20*) gene, as previously described [33]. For further molecular characterization, *E. ruminantium*-positive samples were subjected to a semi-nested PCR assay to amplify a fragment of 720–738 bp of the *map1* gene of *E. ruminantium* [33]. DNA of *E. ruminantium* obtained from naturally infected cattle from Mozambique [34] and ultrapure nuclease-free water were used as positive and negative controls, respectively.

The amplified products were subjected to electrophoresis in 1.8% agarose gels stained with 1% ethidium bromide (Life Technologies^®^, Carlsbad, CA, USA) for 50 min at 90 V. The gels were photographed under ultraviolet light using Image Lab Software (Thermo Fisher Scientific^®^). 

### 2.4. DNA Sequencing and Phylogenetic Analyses

Twenty-seven positive PCR products of the expected size for each assay were purified using the Promega Wizard^®^ PCR and Gel Clean-Up and sequenced in both directions using the same PCR primers (forward and reverse) by Sanger sequencing [35]. 

A maximum likelihood phylogenetic tree was performed by incorporating sequences from the present study and relevant sequences sourced from the GenBank^®^ database (http://www.ncbi.nlm.nih.gov/genbank accessed on 15 March 2024). To ensure the quality of electropherograms and generate consensus sequences, Geneious prime 11.1.3 software was employed. The consensus sequences were subjected to multiple alignments with the sequences selected from GenBank^®^ using MAFFT online software (https://mafft.cbrc.jp/alignment/server/ accessed on 16 March 2024). The best-fit model of nucleotide substitution was determined using jModeltest v.2.1.10 [36] and was set as GTR +G based on the Akaike Information Criterion (AIC). The phylogenetic tree was visualized with FigTree software version 1.4.4.

### 2.5. Statistical Analysis

Data analyses were performed with SPSS Statistics software^®^ (IBM Corp, Armonk, NY, USA, version 26). The chi-square test was used to evaluate significant differences in the infection rate of different species of animals. Odds ratios (ORs), 95% confidence intervals (95% CI), and *p*-values were calculated separately for each variable, and the results were considered significant when *p* ≤ 0.05. Data were compiled and analyzed in Epi Info™ software, version 7.2.3.1 (Centers for Disease Control and Prevention, CDC, USA).

## 3. Results

The *gapdh* gene was consistently amplified from all DNA samples. Overall, 140/530 (26.4%, 95% CI: 22.7–30.4%) animals tested positive for *Ehrlichia* sp. by the *dsb*-PCR assay. Notably, a high occurrence of *Ehrlichia* sp. was observed in dromedary camels (85/155; 54.8%). Among the cattle analyzed, 39/131 (29.8%) tested *Ehrlichia*-positive, while 4/199 (7.0%) goats and 2/45 (4.4%) sheep (Table 1). Dromedary camels (OR: 26.1, *p* < 0.001, χ^2^ = 36.0), cattle (OR: 9.1, *p* < 0.001, χ^2^ = 12.1), and goats (OR: 1.6, *p* = 0.81, χ^2^ = 0.4) were more likely to be infected with *Ehrlichia* sp. than sheep.

Further analysis using *sodB* gene-based PCR revealed that out of the 39 *Ehrlichia*-positive cattle by the *dsb*-PCR assay, 30 (76.9%) also tested positive for the *sodB* gene. However, samples from dromedary camels, goats, and sheep that tested *Ehrlichia*-positive by the *dsb*-PCR assay tested negative by the *sodB*-PCR assay (Table 1). 

Twelve out of the thirty-nine (30.8%) *Ehrlichia*-positive cattle were infested by ticks. However, no significant association was observed between *Ehrlichia* sp. positivity and the presence of ticks (OR = 1.5; 95% CI: 0.6–3.4; *p* = 0.48). Conversely, for dromedary camels, 23/85 (27.1%) identified as *Ehrlichia*-positive animals were infested by ticks, with an association between *Ehrlichia* sp. positivity and tick infestation (OR = 0.16; 95% CI: 0.08–0.32; *p* < 0.001). It is noteworthy that there was no association between *Ehrlichia* positivity and the presence of ticks in goats and sheep.

Moreover, 18 out of 131 (13.7%, 95% CI: 8.3–20.8) cattle tested positive for the *pCS20* gene of *E. ruminantium*. Notably, all positive samples subjected to the *E. ruminantium map1* gene assay tested negative. Among the 18 animals testing positive for *E. ruminantium*, 11 were concurrently infested by ticks. A statistically significant association was observed between *E. ruminantium* positivity in cattle and the presence of ticks (OR = 6.7; 95% CI: 2.3–9.4; *p* = 0.001). 

In total, 28 amplicons were sequenced: eighteen from cattle (nine *Ehrlichia* sp. *dsb* genes, eight *Ehrlichia sodB* genes, and one *E. ruminantium PCS20* gene), four from goats (all *Ehrlichia* sp. *dsb* genes), one from sheep (*Ehrlichia* sp. *dsb* gene), and five from dromedary camels (all *Ehrlichia* sp. *dsb* gene). The BLASTn results for all agents and target genes are shown in Table 2.

**Figure 1:**
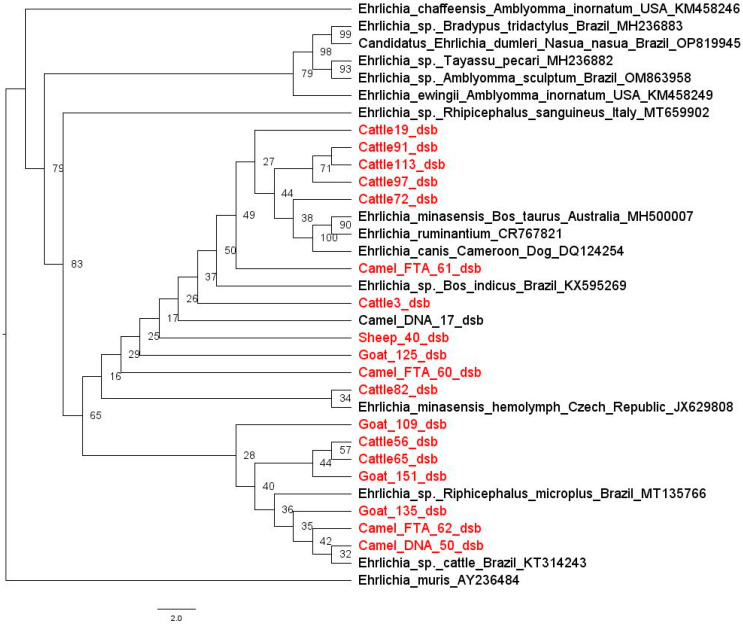
Phylogenetic tree inferred by using maximum likelihood inference and GTR+G evolutionary model based on an alignment of 390 bp of the *dsb* gene. The sequences detected are highlighted in red in the present study. *Ehrilichia muris* was used as the outgroup.

The phylogenetic analysis based on the *Ehrlichia dsb* gene (390 bp alignment) was performed using sequences detected in cattle, goats, sheep, and dromedary camels and selected sequences of *Ehrlichia* sp. obtained from the GenBank^®^ database. The obtained sequences from studied animals grouped with *Ehrlichia* sp., (GenBank^®^ KT314243, MT135766, KX595269) and *E. minasensis* (GenBank^®^ JX629808) sequences detected in ticks from Czech Republic and Brazil are shown in Figure 1.

**Figure 2:**
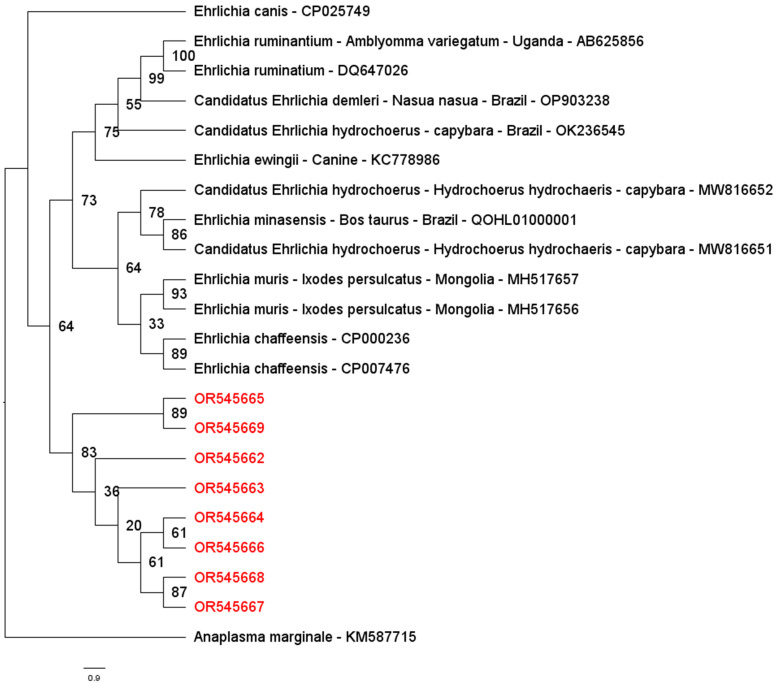
Phylogenetic tree inferred by using maximum likelihood inference and GTR+G evolutionary model based on an alignment of 300 bp of the *sodB* gene. The sequences detected are highlighted in red in the present study. The numbers at the nodes correspond to posterior probability values higher than 50% accessed with 1000 replicates. *A. marginale* was used as outgroups.

The phylogenetic analysis based on the *Ehrlichia sodB* gene (300 bp alignment) was performed using sequences detected in cattle and selected sequences of *Ehrlichia* sp. obtained from the GenBank^®^ database. The obtained sequences from studied animals grouped separately are shown in Figure 2.

**Figure 3:**
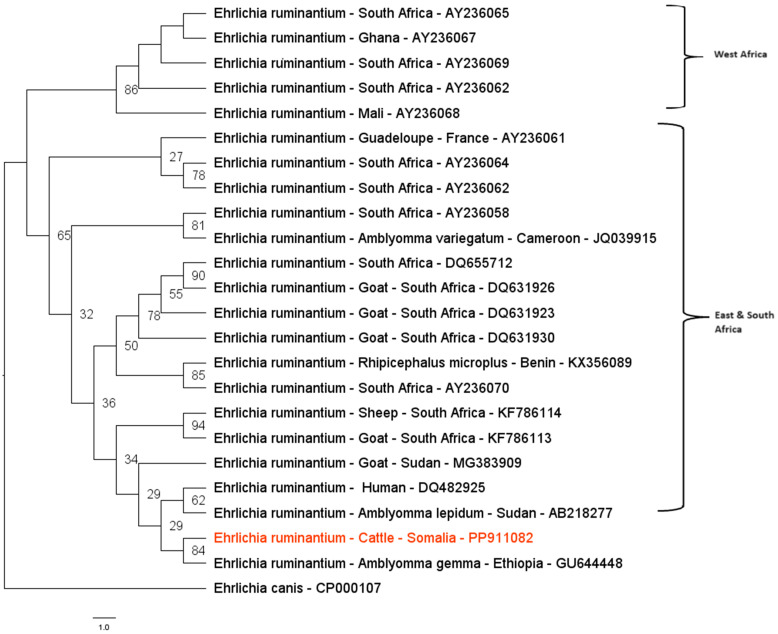
Phylogenetic tree inferred by using maximum likelihood inference and GTR+G evolutionary model based on an alignment of 280 bp of the *E. ruminantium PCS20* gene. The sequences detected are highlighted in red in the present study. The numbers at the nodes correspond to posterior probability values higher than 50% accessed with 1000 replicates. *E. canis* was used as the outgroup.

The phylogenetic analysis based on the *E. ruminantium pCS20* gene (280 bp) was performed using sequences detected in cattle and selected sequences of *E. ruminantium* obtained from GenBank^®^ confirmed the BLASTn analysis. The obtained sequences from studied animals placed within the East and South African clades are shown in Figure 3.

## 4. Discussion

Ehrlichioses are regarded as one of the most economically important TBDs in sub-Saharan Africa and Caribbean islands [2,5]. However, the current economic consequences of ehrlichioses in Somalia remain undetermined, primarily due to the absence of knowledge regarding the occurrence, distribution, and genetic diversity of their causative agents. In this study, we present the first molecular identification of *Ehrlichia* spp. in ruminants and dromedaries from Somalia. To detect the prevalence and spatial distribution of *Ehrlichia* spp., we employed molecular assays targeting *Ehrlichia* spp. based on both the *dsb* and *sodB* genes, along with the species-specific identification of *E. ruminantium* based on the *pCS20* gene specifically for cattle.

The highest prevalence for *Ehrlichia* was observed in dromedaries (54.8%), followed by cattle (29.8%), goats (7%), and sheep (4.4%), respectively. This prevalence differs from previous investigations, such as those conducted in the Caribbean islands, where 385 cattle (19.7%), 340 sheep (13.2%), and 376 goats (3.5%) were positive in a PCR targeting the 16S rRNA gene [17]. In China, a lower prevalence was reported, with 1830 cattle (3.6%), 111 sheep (1.8%), and 270 goats (1.1%) positive in a PCR targeting the 16S rRNA gene [17]. Consistently, our study aligns with Zhang [16] and Qiu [17], reporting consistency in ruminants, where cattle had the highest prevalence. Notably, a study sampling 196 cattle, 200 sheep, and 198 goats from Sudan and using a PCR based on a 16S rRNA gene demonstrated a complete absence of *Ehrlichia* sp. positivity [37]. Factors such as geographic location, climatic conditions, presence of tick vectors, and population dynamics could contribute to these observed differences in prevalence rates. 

The prevalence for *Ehrlichia* in cattle found herein (29.8%) differs from previous investigations, such as the study conducted in apparently healthy dairy cattle in Kenya, where 3.3% of the 306 blood DNA samples analyzed were positive in a PCR based on the 16S rDNA [15]. Conversely, a study in Brazil reported a higher prevalence, with 48.12% of 347 blood samples from beef cattle in the Brazilian Pantanal testing positive in a PCR targeting the *dsb* gene [8]. While our study detected *E. minasensis* and *E. ruminantium* in cattle, *E. minasensis* was identified in cattle from Brazil [8] and *E. minasensis* was reported in Kenya [15].

In this study, we observed occurrences of *Ehrlichia* in goats and sheep of 7% and 4.4%, respectively. The prevalence found in our study differs from that reported in other regions, such as the Caribbean islands, where sheep showed a prevalence of 13.2% and goats showed 3.5% [16]. Additionally, studies conducted in China reported a prevalence of 1.8% in sheep and 1.1% in goats [17], with both studies showing a higher prevalence in sheep than in goats. Herein, while *E. minasensis* was identified in both goats and sheep, the Caribbean study found *E. canis, E. ovina, Ehrlichia* sp. (BOV2010, UFMG-EV), and *E. ruminantium*, based on identities (98–100%) inferred from 16S rRNA and *gltA* genes [16]. A study from China reported *E. canis* in goats based on the *gltA* identity (98%) [17]. Studies in Malawi reported *E. ruminantium* and *E. canis* detection based on 16S rDNA and *groEL* genes [20]. Additionally, a study in Italy detected *E. canis* in sheep and goats based on the 16S rRNA gene [19].

Out of the 155 dromedaries’ blood samples analyzed, 54.8% were found positive for the *Ehrlichia* sp. *dsb* gene. This prevalence differs from previous investigations. For instance, a study conducted on 296 apparently healthy dromedaries in Kenya reported an occurrence of 14.5% for ‘*Candidatus* Ehrlichia regneryi’ using a PCR based on a 16S rDNA gene [21]. Similarly, a study in Saudi Arabia reported 26% positivity among 100 blood samples from apparently healthy dromedaries testing positive for ‘*Candidatus* Ehrlichia regneryi’ in a PCR targeting the 16S rRNA and *groEL* genes [23]. Herein, we identified *E. minasensis* based on the *Ehrlichia* sp. *dsb* gene, with identity ranging from 98.7 to 100%.

The observed variations in *Ehrlichia* prevalence among the studied animal populations in the present study compared to other studies underscore regional and species-specific differences. Factors like geographic variations, climate conditions influencing tick distribution, the health status of sampled populations, the genetic diversity of *Ehrlichia* strains, and differences in tick vectors and host species contribute to these inconsistencies. These multifaceted factors underscore the complexities of tick-borne diseases, emphasizing the need for comprehensive, context-specific investigations to understand *Ehrlichia* infection dynamics.

Subsequently, after confirming positive results for the *Ehrlichia* sp. *dsb* gene, further characterization was conducted by testing positive samples for the *sodB* gene. Of the 39 positive cattle samples, 30 (76.9%) were positive for the *sodB* gene, indicating a substantial prevalence of *Ehrlichia* spp. infection. However, samples from goats, sheep, and dromedaries that tested positive for *Ehrlichia* spp. based on the *dsb* gene were negative in the *sodB* gene-based assays. This disparity suggests potential variations in *Ehrlichia* strains or species infecting different hosts, underscoring the need for further molecular investigations to elucidate diversity and host-specificity. Alternatively, negative *sodB* results in other animals may be due to assay sensitivity, potentially indicating bacteremia levels below detection limits. Furthermore, partial *dsb* and *sodB* gene sequences from cattle showed inconsistencies in BLAST analysis, suggesting that these sequences may represent different regions of the *dsb* gene of *Ehrlichia* spp. It is also important to note that fewer *sodB* sequences are available in GenBank compared to *dsb* sequences, which may account for some of the observed discrepancies.

Recent studies have unveiled a novel pathogen, *E. minasensis*, initially reported in Canada [7] and Brazil [38] and subsequently identified in various countries, including Brazil [8], France [11], Pakistan [12], Ethiopia [13], South Africa [14], and Kenya [15]. Molecular analyses have revealed the genetic similarity of these strains, suggesting their evolution from a diverse clade within *E. canis* [39]. Phenotypic and genotypic differences from *E. canis* are attributed to the pathogen’s adaptation to new hosts including ruminants and tick vectors [16]. This study detected *E. minasensis* DNA in all animal species studied, including cattle, goats, sheep, and dromedaries. Notably, to the best of the authors’ knowledge, this study revealed, for the first time, the presence of *E. minasensis* in sheep, goats, and dromedaries globally. Such identification was based on *Ehrlichia* sp. *dsb* gene sequencing, exhibiting a high degree of identity (98.5–100%) and the phylogenetic positioning of the detected genotypes.

*Ehrlichia minasensis* has been identified in several tick species, including *R. appendiculatus, R. evertsi eversi, R. sanguineus*, *Amblyomma* sp. [14], *R. microplus* [40], and *Hyalomma* sp. [12]. The transstadial transmission of *E. minasensis* has been demonstrated specifically in *R. microplus* [26]. Considering that this pathogen was found in different tick species, including *Amblyomma* sp. and *Hyalomma* sp., which were found to be parasitizing the studied animals as previously reported for cattle by Ferrari [28], this emphasizes the need for further investigation into the role of these ticks in the transmission dynamics of *E. minasensis* and its prevalence in various livestock species. Moreover, *E. minasensis* infection in cattle, as demonstrated experimentally, presents clinical signs including fever, lethargy, anorexia, anemia, leukopenia, thrombocytopenia, and bleeding, with symptoms resembling chronic canine ehrlichiosis [41,42]. These findings underscore the importance of exploring its prevalence in different livestock species and understanding its impact on animal health.

In the present study, 13.7% of cattle were positive for *E. ruminantium* based on the *pCS20* gene. This prevalence is notably consistent with findings in Maputo Province, Mozambique, where 15% of 210 cattle samples tested positive [34], and in Gambia, where 16.6% of 145 adult *Amblyomma variegatum* ticks collected from indigenous cattle were positive [33]. Conversely, a study in Kenya, utilizing RLB analysis on 453 calves, reported a considerably lower prevalence of 0.4% [43]. Similarly, in Ethiopia, 0.6% of 457 blood samples from cattle across four localities tested positive for the *pCS20* gene of *E. ruminantium* [44]. The endemic stability of heartwater in most sub-Saharan African countries, as highlighted by Deem [45], contributes to generally low infection rates in ticks within the field [46]. Infected animals often remain asymptomatic, harboring the pathogen at minimal levels [44]. Given the intracellular localization of *E. ruminantium* predominantly in endothelial cells and its periodic presence in the bloodstream, the observed low prevalence rates based on PCR assays from blood samples align with expectations [47,48]. Notably, none of the samples positive for the *pCS20* gene were positive in the PCR assays targeting the *map1* gene, likely due to low bacteremia levels below the detection limit of the molecular assays. The *pCS20* gene, specific to *E. ruminantium*, is more sensitive to detection, while the *map1* gene is commonly used for diagnosing and characterizing various genotypes of the pathogen [49].

Heartwater occurs in wild and domestic ruminants where the tick vectors, primarily *Amblyomma* species, are present, predominantly in Africa. *Amblyomma variegatum* and *Amblyomma hebraeum* represent the two most important tick vectors associated with the distribution of disease in Africa [50]. In Somalia, *A. variegatum* has been identified in cattle, goats, sheep, and dromedaries [27,51], but *A. hebraeum*, to the best of our knowledge, has not been reported yet. Notably, the ticks collected from the studied animals did not include *A. variegatum* and *A. hebraeum*, suggesting the possible transmission of *E. ruminantium* by ticks other than these two species. Further investigation is necessary to understand the significance of this pathogen for cattle health and to identify the vectors responsible for its transmission in Somalia.

In this study, only one cattle sample was successfully sequenced for the *E. ruminantium PCS20 gene*. The partial sequence obtained showed a high identity of 98.5% with a sequence identified in *A. variegatum* ticks from Uganda (MK371032). Phylogenetic analysis of the *PCS20* gene indicated that the positive case in this study closely resembled *E. ruminantium* isolated from *A. gemma* ticks collected in Ethiopia (GU644448), placing our sequences within the East and South Africa clade.

*Ehrlichia minasensis* and *E. ruminantium* are the only species of *Ehrlichia* known to naturally infect cattle [18]. The cattle sampled in this study showed a 16.3% rate of co-infection by *E. minasensis* and *E. ruminantium*. The coexistence of these pathogens may contribute to heightened disease complexity and severity, potentially influencing clinical manifestations, immune responses, and overall health outcomes in the infected cattle [13]. This underscores the necessity of considering multiple *Ehrlichia* spp. in the diagnostic and management strategies for diseases affecting cattle populations. Further research is needed to clarify the specific interactions between *E. minasensis* and *E. ruminantium*, shedding light on their implications for animal health.

It is important to acknowledge a key limitation regarding the construction of the phylogenetic tree. Due to sequencing constraints, we were only able to successfully sequence one sample for the *E. ruminantium PCS20* gene. While this limitation narrows the scope of our analysis, it also underscores the need for further research with larger sample sizes to provide a more comprehensive understanding. Nonetheless, our study’s focus on exploring the genetic diversity within *E. ruminantium* remains a notable strength, pointing toward paths for future research to explore deeper into the evolutionary dynamics of this pathogen within our target population.

In conclusion, this study represents a significant step toward understanding the prevalence and genetic diversity of *Ehrlichia* sp. in ruminants and dromedaries from the Lower Shabelle region of Somalia. The detection of *Ehrlichia* sp. in 26.4% of the sampled animals underscores the substantial prevalence of this infection in the studied population. This is the first report of *E. minasensis* in cattle from Somalia and the first report of this agent in sheep, goats, and dromedaries worldwide. Furthermore, the identification of *E. ruminantium* in 13.7% of the samples adds valuable insights into the epidemiology of heartwater disease in Somalia.

## Figures and Tables

**Table 1:** Prevalence of *Ehrlichia* spp.

	*dsb* Gene		*sodB* Gene	
Animal Species	+/n	95% CI	+/n	95% CI
Dromedary	85/155	54.8 (46.7–62.8%)	0/85	0
Cattle	39/131	29.8%, (22.1–38.4%)	30/39	76.9 (60.7–88.9%)
Goat	4/199	7.0% (3.9–11.5%)	0/4	0
Sheep	2/45	4.4% (0.5–15.2%)	0/2	0

**Table 2:** Percentage of BLASTn-associated identity of sequences of *Ehrlichia* spp. detected in ruminants and dromedary camels from Somalia.

Agents	Animal ID	Accession Number	Target Gene	Query Cover	E-Value	Identity	GenBank^®^ Accession Number
*Ehrlichia* sp.	Cattle 3	*PQ041218*	*dsb*	100%	2 × 10^−161^	99.4%	*E. minasensis* collected from *Rhipicephalus microplus* from Panama (OR711253)
*Ehrlichia* sp.	Cattle 19	*PQ041219*	*dsb*	98%	3 × 10^−164^	99.1%	*E. minasensis* collected from *Bos Taurus* from Australia (MH500007)
*Ehrlichia* sp.	Cattle 56	*PQ041220*	*dsb*	100%	2 × 10^−165^	100%	*E. minasensis* collected from *Rhipicephalus* sp. from Pakistan (OQ627010)
*Ehrlichia* sp.	Cattle 65	*PQ041221*	*dsb*	100%	2 × 10^−166^	100%	*E. minasensis* collected from *Bos Taurus* from Philippines (LC641910)
*Ehrlichia* sp.	Cattle 72	*PQ041222*	*dsb*	100%	1 × 10^−158^	99.4%	*E. minasensis* collected from *Rhipicephalus microplus* from Panama (OR711253)
*Ehrlichia* sp.	Cattle 82	*PQ041223*	*dsb*	100%	4 × 10^−167^	99.1%	*E. minasensis* collected from *Bradypus variegatus* from Brazil (MT212415)
*Ehrlichia* sp.	Cattle 91	*PQ041224*	*dsb*	100%	3 × 10^−164^	99.7%	*E. minasensis* collected from *Rhipicephalus microplus* from Panama (OR711253)
*Ehrlichia* sp.	Cattle 97, 113	*PQ041225, PQ041226*	*dsb*	98%	6 × 10^−166^	99.4%	*E. minasensis* collected from *Bos Taurus* from Australia (MH500007)
*Ehrlichia* sp.	Goat 109	*PQ084567*	*dsb*	100%	4 × 10^−137^	99.6%	*E. minasensis* collected from *Bradypus variegatus* from Brazil (MT212415))
*Ehrlichia* sp.	Goat 125	*PQ084568*	*dsb*	96%	0	99.3%	*E. minasensis* collected from *Bradypus variegatus* from Brazil (MT212415)
*Ehrlichia* sp.	Goat 135	*PQ084569*	*dsb*	94%	0	99.5%	*E. minasensis* collected from *Rhipicephalus microplus* from Czech Republic (JX629808)
*Ehrlichia* sp.	Goat 151	*PQ084570*	*dsb*	100%	8 × 10^−188^	98.9%	*E. minasensis* collected from *Bradypus variegatus* from Brazil (MT212415)
*Ehrlichia* sp.	Sheep 40	*PQ084571*	*dsb*	100%	1 × 10^−161^	100%	*E. minasensis* collected from *Rhipicephalus microplus* from Czech Republic (JX629808)
*Ehrlichia* sp.	Dromedary FTA 60	*PQ084562*	*dsb*	100%	2 × 10^−114^	100%	*E. minasensis* collected from *Rhipicephalus* sp. from Pakistan (OQ627010)
*Ehrlichia* sp.	Dromedary FTA 61	*PQ084563*	*dsb*	100%	1 × 10^−151^	100%	*E. minasensis* collected from *Rhipicephalus* sp. from Pakistan (OQ627010)
*Ehrlichia* sp.	Dromedary FTA 62	*PQ084564*	*dsb*	100%	2 × 10^−159^	100%	*E. minasensis* collected from *Bos Taurus* from Australia (MH500007)
*Ehrlichia* sp.	Dromedary DNA 17	*PQ084565*	*dsb*	100%	9 × 10^−164^	99.4%	*E. minasensis* collected from *Rhipicephalus* sp. from Pakistan (OQ627010)
*Ehrlichia* sp.	Dromedary DNA 50	*PQ084566*	*dsb*	100%	3 × 10^−153^	98.7%	*E. minasensis* collected from *Bos Taurus* from Philippines (LC641910)
*Ehrlichia* sp.	Cattle 3, 19	*OR545662, OR5456653*	*sodB*	100%	2 × 10^−74^	85.4%	*Ehrlichia* sp. strain H7 collected from Capybara from Brazil (MW816653)
*Ehrlichia* sp.	Cattle 56	*OR545664*	*sodB*	100%	1 × 10^−77^	85.4%	*Ehrlichia* sp. strain H7 collected from Capybara from Brazil (MW816653)
*Ehrlichia* sp.	Cattle 65	*OR545665*	*sodB*	100%	4 × 10^−57^	83.8%	*E. ewingii* collected from dog from USA (KC778986)
*Ehrlichia* sp.	Cattle 72, 82	*OR545666, OR545667*	*sodB*	99%	3 × 10^−68^	83.4%	*Ehrlichia* sp. strain H7 collected from horse from USA (KJ434180)
*Ehrlichia* sp.	Cattle 91	*OR545668*	*sodB*	100%	7 × 10^−70^	83.4%	*E. ruminantium* collected from Senegal (DQ647026)
*Ehrlichia* sp.	Cattle 113	*OR545669*	*sodB*	99%	3 × 10^−69^	83.1%	*E. ruminantium* collected from Senegal (DQ647026)
*E. ruminantium*	Cattle 79	*PP911082*	*PCS20*	100%	4 × 10^−132^	98.5%	*E. ruminantium* collected from *Amblyomma variegatum* from Uganda (MK371032)

## Data Availability

The data underpinning the conclusions of this study can be obtained from the corresponding authors upon reasonable request.
